# Shewanella algae as a Rare Cause of Bullous Cellulitis and Sepsis: The First Reported Case in Lithuania

**DOI:** 10.7759/cureus.98958

**Published:** 2025-12-11

**Authors:** Evelina Bucionyte, Gabija Dragunaite, Arūnas Petkevičius, Asta Dambrauskiene

**Affiliations:** 1 Department of Skin and Venereal Diseases, Medical Academy, Lithuanian University of Health Sciences (LSMU), Hospital of Lithuanian University of Health Sciences Kaunas Clinics, Kaunas, LTU; 2 College of Medicine, Lithuanian University of Health Sciences (LSMU), Kaunas, LTU; 3 Department of Laboratory Medicine, Medical Academy, Lithuanian University of Health Sciences (LSMU), Hospital of Lithuanian University of Health Sciences Kaunas Clinics, Kaunas, LTU

**Keywords:** baltic sea, bullous cellulitis, sepsis, shewanella algae infection, type 2 diabetes

## Abstract

*Shewanella algae* is an emerging marine bacterium capable of causing serious infections in immunocompromised individuals. We report the first documented case in Lithuania of bullous cellulitis caused by *S. algae*, complicated by necrotizing soft-tissue infection and ultimately requiring leg amputation. To determine whether similar cases had been described earlier in Lithuania, a structured literature review was conducted. We searched PubMed/MEDLINE, Embase, Scopus, Web of Science, and Google Scholar by combining the terms “Shewanella algae,” “Shewanella,” “Baltic Sea,” “wound infection,” “necrotizing infection,” “sepsis,” “case report,” and “Lithuania.” No date limits were set, and all titles and abstracts were screened.

A 74-year-old woman with poorly controlled type 2 diabetes and recent seawater exposure presented with acute right leg pain and skin lesions. Blood and wound cultures confirmed *S. algae* infection. Despite broad-spectrum antibiotics and intensive care, the patient’s condition deteriorated, requiring below-knee and later above-knee amputation. This case emphasizes the aggressive clinical course *S. algae* can take in vulnerable patients and highlights the importance of early recognition and multidisciplinary management of severe skin and soft tissue infections.

## Introduction

*Shewanella algae* is a saprophytic, Gram-negative bacillus found predominantly in marine and brackish environments [1]. Human infection typically results from direct contact between seawater and compromised skin, and the risk is substantially higher in individuals with metabolic comorbidities, particularly diabetes mellitus, due to impaired wound healing and reduced local immune defense [2]. Clinically, *S. algae* most commonly causes skin and soft-tissue infections (SSTIs) such as cellulitis and bullous lesions, and in severe cases may progress to necrotizing fasciitis or bacteremia [3,4]. High-risk patients with chronic wounds or recent seawater exposure can deteriorate rapidly because of the organism's invasive potential that enables deep tissue involvement and fulminant soft-tissue destruction. Diagnosis relies on microbiological confirmation from wound swabs or blood cultures, and although many strains remain susceptible to standard antimicrobials, drug resistance and the need for urgent surgical intervention have been increasingly reported [2,5].

The paper describes the first documented case in Lithuania of *S. algae* infection presenting as an acute bullous cellulitis, which rapidly evolved into a necrotizing soft-tissue infection, finally necessitating amputation of the lower limb.

## Case presentation

We present the case of a 74-year-old woman who arrived at the emergency department with an abrasion on her right heel and severe leg pain, rated 9/10 on the Visual Analogue Scale (VAS). Her medical history included poorly controlled type 2 diabetes mellitus for 30 years (HbA1c 10.1% on June 17, 2024) (Table 1). The patient also had a history of secondary hypertension, chronic atrial fibrillation, and arthritis. She had no known allergies or previous surgeries.

**Table 1:** Trend of C-reactive protein (CRP) levels during hospitalization

Date	CRP (mg/L, reference range <5.0)	Clinical Interpretation
August 4, 2024	144.4	Elevated inflammatory marker at admission
August 5, 2024	332.4	Marked increase, indicating infection progression
August 6, 2024	494.7	Further rise, consistent with severe inflammation/sepsis
August 9, 2024	201.1	Decline associated with clinical improvement
August 26, 2024	51.2	Significant reduction after therapy
August 27, 2024	111.9	Mild rebound of inflammation
August 30, 2024	212.3	Recurrent inflammation prior to surgery

Between July 22 and 25, 2024, the patient was on vacation and swam in the Baltic Sea. Although no new trauma occurred, she had a chronic diabetic heel wound, likely serving as the portal of entry. On August 4, 2024, she developed acute right leg pain and presented to the emergency department. Due to an existing heel wound and elevated C-reactive protein (CRP) level of 144.4 mg/L, she was urgently hospitalized in the Endocrinology Department at Kaunas Clinics, where empirical antibiotic therapy with intravenous cefuroxime 1.5 g three times daily and oral metronidazole 500 mg three times daily was initiated.

Shortly after admission, her condition worsened, with the development of respiratory failure, a paroxysm of tachysystolic atrial fibrillation, and suspected sepsis, prompting transfer to the intensive care unit (ICU). CRP increased markedly to 332.4 mg/L on August 5. Antibiotic therapy was escalated to intravenous piperacillin/tazobactam 4.5 g four times daily. As CRP continued to rise to 494.7 mg/L on August 6, intravenous vancomycin 1 g once daily was added.

Blood and wound cultures taken on August 4, 2024, from the right leg abrasion yielded *S. algae*. An isolated *S. algae *strain was identified using matrix-assisted laser desorption/ionization (MALDI) mass spectrometry (MALDI Biotyper Sirius; Bruker Daltonics, Bremen, Germany). *S. algae* was susceptible (increased exposure) to piperacillin/tazobactam, ceftazidime, cefepime, and ciprofloxacin; susceptible to amikacin and meropenem; and resistant to imipenem. Antimicrobial susceptibility was determined by the disk diffusion method. The European Committee on Antimicrobial Susceptibility Testing (EUCAST) has not established specific clinical breakpoints for *S. algae*. In this case, inhibition zones were interpreted using EUCAST breakpoints for *Pseudomonas* spp. Based on these findings, vancomycin was discontinued, and treatment with piperacillin/tazobactam was continued.

**Figure 1:**
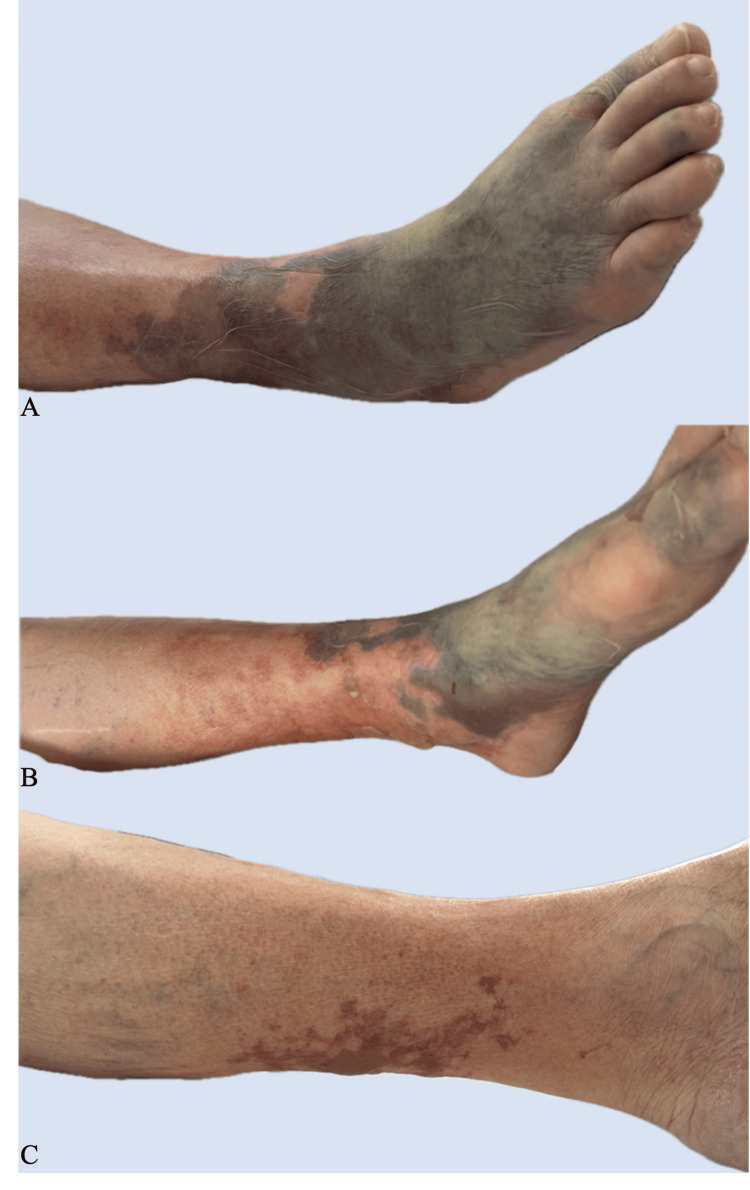
Confluent bullous lesions containing hemorrhagic and, in some areas, serous fluid on the skin of the right foot and calf (A, B). A single tense serous-filled bullous lesion surrounded by erythema is seen on the dorsal surface of the left calf (C).

Dermatologic examination revealed multiple confluent bullous lesions filled with hemorrhagic fluid and, in some areas, serous fluid on the skin of the right foot and calf. The affected leg was cold on palpation and showed signs of impaired microcirculation. Additionally, a single tense bullous lesion filled with serous fluid and surrounded by erythema was noted on the dorsal surface of the left calf. No signs of necrosis were present (Figure 1). A diagnosis of bullous cellulitis was confirmed.

As the patient’s condition improved and inflammatory markers decreased (CRP 201.1 mg/L) on August 9, 2024, she was transferred back to the Endocrinology Department. Shortly after transfer, early signs of tissue compromise appeared. Although the initial bullae improved, the right leg developed worsening trophic skin changes, persistent cold tissue, impaired perfusion, and a non-healing ulcer, all consistent with evolving necrosis. Peripheral pulses were weak. Bilateral leg angiography confirmed significant ischemia, and an attempted revascularization was unsuccessful.

Rising inflammatory markers, persistent infection, and angiographic findings supported the decision to perform a right below-knee amputation. At this point, the left leg showed no ischemia: it was warm, pulses were not documented as absent, and no necrotic changes were seen. Thus, no proximal spread of infection or ischemia was detectable prior to the initial surgery.

The patient initially refused the procedure. Following clinical deterioration and identification of retroperitoneal bleeding on a CT angiogram, a right below-knee amputation was performed on August 23, 2024. From August 23 to 27, the patient remained in the ICU. Elevated inflammatory markers prompted ongoing evaluation for infection sources. Because the isolate was susceptible to piperacillin/tazobactam and inflammatory markers had previously improved, the antibiotic was continued initially in accordance with a stepwise, susceptibility-guided management strategy.

After stabilization, she was transferred back to the Endocrinology Department. Initially, inflammatory markers declined, but between August 27 and 28, CRP rose again from 51.2 mg/L to 111.9 mg/L. In response to this secondary inflammatory rise, antibiotic therapy was escalated to meropenem 1 g three times daily. Despite escalation, CRP increased to 212.3 mg/L by August 30. A multidisciplinary team concluded that the most likely source of persistent inflammation was ongoing necrosis in the right leg.

Although the below-knee amputation addressed necrotic tissue visible at that time, further necrosis extended above the prior amputation line over subsequent days. This represented a new proximal progression, not detectable before the first surgery. Persistent ischemia, non-viable tissue, and rising inflammatory markers despite meropenem justified a right above-knee amputation, which was performed on August 31, 2024. This case highlights a severe soft-tissue infection complicated by *S. algae*, the first bacteremia caused by *S. algae* identified at our hospital.

## Discussion

Epidemiology and microbiological characteristics of *S. algae*

In recent years, the number of infections caused by *Shewanella* species has been on the rise [4]. Among them, *S. algae* appears to be more virulent than other *Shewanella* species [6]. *Shewanella* species have been isolated from oysters, abalone, and clams [7], and the ability of these bacteria to tolerate wide temperature ranges (4°C-42°C) and salinities up to 6% NaCl [7] enables them to survive not only in tropical waters but also in temperate brackish environments. Most reported infections come from warmer countries [4]. However, during the summer, the Baltic Sea reaches temperatures within the range for optimal growth of *S. algae*, making it a possible reservoir. Ecological adaptability provides an explanation for how an infection might be contracted through exposure in the Baltic Sea. In our patient, a chronic diabetic heel wound provided a susceptible portal of entry, and recent seawater exposure provided a chance for inoculation with a species recognized as more virulent compared with other *Shewanella* strains [6]. Together, these factors explain the patient's rapid progression from bullous cellulitis to necrotizing soft-tissue infection.

Although *Shewanella* can infect individuals of all ages, people over 60 years old are particularly vulnerable. The male-to-female ratio of infection is approximately 2.84:1, likely due to greater male participation in marine-related occupations and activities such as fishing and diving [2]. Virulence factors of *S. algae* include hemolytic activity, enzymatic production, and biofilm formation [8]. *Shewanella* spp. also possess mechanisms that enable them to assimilate environmental carbohydrates, demonstrating their exceptional adaptability [7].

Clinical presentation of *S. algae*

The primary routes of *Shewanella* infection include exposure of open wounds to seawater, trauma-related contamination of injured tissue, ingestion of contaminated seafood (leading to gastrointestinal infection), and entry through the ear canal. Clinical manifestations of *S. algae* infection can be categorized into eight groups: ear, nose, and throat disorders; central nervous system infections; respiratory infections; cardiovascular diseases; bloodstream infections (bacteremia, septicemia); intra-abdominal infections; bone and joint infections; and SSTIs. SSTIs - including cellulitis, abscess formation, and necrotizing fasciitis - are the most commonly reported clinical presentations [4]. Rare but serious complications have also been documented, including necrotizing soft-tissue infections, aortic aneurysm rupture, peritonitis, and endocarditis [9,10]. Bacteremia is a complication in at least 28% of reported cases [11]. In a study of 27 cases of *Shewanella*-related SSTIs, 82% involved infections of the extremities, with over half associated with chronic leg ulcers [12]. Although rare, infections in immunocompetent individuals have been observed. For example, several young men undergoing U.S. Naval Special Warfare (NSW) training developed infections despite having no comorbidities. Prolonged exposure to marine environments, intense physical training, sleep deprivation, and environmental stress were proposed as contributing factors [13]. Another report describes an immunocompetent woman whose only identified risk factors were obesity and peripheral arterial disease; she contracted *Shewanella* despite lacking any known exposure to seawater [14]. The first documented case of* S. algae* infection associated with the Baltic Sea was reported in 2025, raising the possibility that such infections may become more prevalent in Scandinavian countries [15]. This underscores the importance of careful diagnostic consideration, especially in patients with a history of bathing in the Baltic Sea.

Diagnostic evaluation of *S. algae*

*S. algae* infection should be suspected in patients with relevant clinical symptoms and a history of seawater exposure. Laboratory testing is essential for diagnosis. The organism can be isolated from a variety of clinical specimens, including blood, sputum, urine, intra-abdominal fluid, stool, and bile. Most commonly, *S. algae* is isolated from blood cultures and wound swabs [4]. Isolation from ear swabs has also been reported [16]. Identification is generally straightforward: *Shewanella* are Gram-negative, motile bacilli that form tan-colored, beta-hemolytic colonies on blood agar. It is often difficult to identify *Shewanella* species using biochemical tests, but 16S rRNA gene sequencing and MALDI time-of-flight mass spectrometry are widely used for the identification and have improved the detection of *Shewanella* species as pathogenic microorganisms [17].

Management and treatment of *S. algae*

Because of the rarity and diversity of the infections caused by *S. algae*, there is no standard treatment protocol yet. *S. algae* may also be present with other pathogens, contributing to polymicrobial infections [18]. The antibiotic susceptibility pattern depends on the genetic background of the *Shewanella* strain. Although there is a variation in resistance, *Shewanella* spp. are generally sensitive to third- and fourth-generation cephalosporins, carbapenems, β-lactamase inhibitor combinations, aminoglycosides, chloramphenicol, erythromycin, aztreonam, and quinolones [11,19]. The resistance mechanism involves mainly genes, and several such genes have been identified in *Shewanella* spp. Soft-tissue infections due to *S. algae* are treated with β-lactams, aminoglycosides, and quinolones. Broad-spectrum antibiotics such as ceftazidime have been used successfully to halt infection progression [10]. In this case, the isolate was resistant to imipenem but susceptible to meropenem, reflecting the variability at a species level with respect to carbapenem susceptibility among *Shewanella* spp. Clinically, this therefore points to the necessity for individualized susceptibility testing rather than assuming uniform carbapenem activity, since imipenem resistance can limit empirical use of carbapenem therapy and require the use of other active agents such as meropenem.

## Conclusions

*S. algae *is an emerging human pathogen associated with aquatic environments and an increasingly serious cause of severe infections, especially among immunocompromised patients and those with chronic comorbidities. The organism thrives over a wide temperature and salinity range, an important factor in its global distribution and its clinical relevance. The most common clinical presentation is as an SSTI, often progressing to more serious conditions, including necrotizing fasciitis, bacteremia, and septic shock. Infection in individuals with normal immune status is rare, while exposure to environmental conditions, especially through open wounds in marine settings, is a major risk factor.

Our case illustrates how rapidly *S. algae* infection can deteriorate in a high-risk host: despite early recognition, prompt broad-spectrum antibiotic therapy, and intensive care, the patient progressed to extensive necrotizing soft-tissue infection, requiring both below-knee and ultimately above-knee amputation. This underlines the aggressive clinical course that *S. algae* can take in vulnerable individuals. This case illustrates that, even with early antibiotics, *S. algae* infection can rapidly progress and may necessitate urgent surgical intervention, including major limb amputation. In addition, due to the lack of standardized treatment guidelines, correct microbiological diagnosis and, subsequently, timely initiation of an appropriate antibiotic are of particular importance. Growing cases of *S. algae* infections in temperate regions, including the Baltic Sea region, strongly call for heightened clinical awareness among patients with a history of seawater exposure. Further studies are necessary to elucidate better the pathogen's virulence mechanisms, resistance patterns, and effective management options.
